# A Parallel Framework with Block Matrices of a Discrete Fourier Transform for Vector-Valued Discrete-Time Signals

**DOI:** 10.1155/2015/348517

**Published:** 2015-09-15

**Authors:** Pablo Soto-Quiros

**Affiliations:** ^1^Escuela de Matemáticas, Instituto Tecnológico de Costa Rica, Apartado 159-7050, 30101 Cartago, Costa Rica; ^2^Centre for Industrial and Applied Mathematics, University of South Australia, Adelaide, SA 5095, Australia

## Abstract

This paper presents a parallel implementation of a kind of discrete Fourier transform (DFT): the vector-valued DFT. The vector-valued DFT is a novel tool to analyze the spectra of vector-valued discrete-time signals. This parallel implementation is developed in terms of a mathematical framework with a set of block matrix operations. These block matrix operations contribute to analysis, design, and implementation of parallel algorithms in multicore processors. In this work, an implementation and experimental investigation of the mathematical framework are performed using MATLAB with the Parallel Computing Toolbox. We found that there is advantage to use multicore processors and a parallel computing environment to minimize the high execution time. Additionally, speedup increases when the number of logical processors and length of the signal increase.

## 1. Introduction

Let *l* ^2^(*ℤ*_*n*_, *ℂ* ^*d*^) be the space of vector-valued discrete-time signals with *n* samples, where each sample is a complex vector of length *d*. The vector-valued discrete-time signals are used very often in several applications in signal processing and electrical engineer, for example, vector quantization of images [1], time-frequency localization with wavelets [2], image coding [3], vector filter bank theory [4], linear time-dependent MISO [5], and analysis of MMSE estimation for compressive sensing of block sparse signals [6].

Now, to analyze the spectra of vector-valued discrete-time signals, a novel tool was developed, and it is called* vector-valued DFT* [7, 8]. This transform has applications in vector analysis in complex, quaternion, biquaternion, and Clifford algebras [8]. Additionally, the vector-valued DFT is used in digital signal processing, for example, the study of new complex valued constant amplitude zero autocorrelation (CAZAC) signals [9], which serve as coefficients for phase coded waveforms with prescribed vector-valued ambiguity function behavior, which is relevant in light of time-frequency analysis, vector sensor, and MIMO technologies [7].

The following paper presents a parallel framework of the vector-valued DFT. The major contributions of this paper are summarized as follows:

1. The construction of a new mathematical structure for the vector-valued DFT using block matrix theory such that it allows a parallel implementation in multicore processors.
2. Reducing the elapsed time to compute the vector-valued DFT of a vector-valued discrete-time signal using parallel computing through aforementioned new mathematical framework.

This new framework is developed with a set of block matrix operations, for example, Kronecker product, direct sum, stride permutation, vec operator, and vec inverse operator (see Section 2.1 for details). These block matrix operations contribute to analysis, design, and implementation of parallel algorithms in multicore processors [10–12]. This mathematical framework is inspired in the matrix representation of the Cooley-Tukey fast Fourier transform (FFT) algorithm for complex discrete-time signals, corresponding to the decomposition of the transform size *n* into the product of two factors *r* and *s*, which is developed in [10, 12, 13].

The present paper is organized as follows.  Section 2 explains a mathematical background about block matrix operations and discrete Fourier transform.  Section 3 defines the concept of vector-valued DFT for vector-valued discrete-time signals.  Section 4 develops a mathematical framework of vector-valued DFT in terms of block matrix operations for vector-valued discrete-time signals with length *n* = *rs*. This mathematical framework contributes to implementation of parallel algorithms.  Section 5 explains an implementation and experimental investigation of this mathematical framework using parallel computing in multicore processors with MATLAB. Finally, some conclusions are presented in Section 6.

Throughout the paper, the following notations are used. *ℤ*_*n*_ = {0,1,…, *n* − 1} is the additive group *ℤ* of integers modulo *n*, *ℂ* ^*m*×*n*^ is the matrix space of *m* rows and *n* columns with complex numbers entries and *ℂ* ^*n*^ = *ℂ* ^*n*×1^. The rows and columns of **A** ∈ *ℂ* ^*m*×*n*^ are indexed by elements of *ℤ*_*m*_ and *ℤ*_*n*_, respectively. **A**(*j*, *k*), **A**(*j*, :), **A**(:, *k*), and **A** ^*T*^ represent entry (*j*, *k*), row *j*, column *k*, and transpose matrix of **A**, respectively. **I**_*n*_ ∈ *ℂ* ^*n*×*n*^ is identity matrix.

## 2. Background

### 2.1. Block Matrix Operations

A block matrix **A** ∈ *ℂ* ^*mp*×*nq*^ with *m* row partitions and *n* column partitions and a block vector **x** ∈ *ℂ* ^*mp*^ with *m* row blocks are defined as

(1) A=A0,0⋯A0,n−1⋮⋱⋮Am−1,0⋯Am−1,n−1,x=x0⋮xm−1,

respectively, where **A**_*j*,*k*_ ∈ *ℂ* ^*p*×*q*^ designates (*j*, *k*) block and **x**_*j*_ ∈ *ℂ* ^*p*^ designates *j* block. In this paper, the following block matrix operations are used: Kronecker product, direct sum, stride permutation, vec operator, and vec inverse operator.

The Kronecker product of two matrices **A** ∈ *ℂ* ^*m*×*n*^ and **B** ∈ *ℂ* ^*p*×*q*^ is defined as **A** ⊗ **B** ∈ *ℂ* ^*mp*×*nq*^ and it replaces every entry (*j*, *k*) of **A** by the matrix **A**(*j*, *k*)**B**. In the special case **A** = **I**_*n*_, it is called parallel operation [12].

The direct sum of *n* matrices constructs a block diagonal matrix from a set of matrices, that is, for {**C**_*k*_}_*k*∈*ℤ*_*n*__, such that **C**_*k*_ ∈ *ℂ* ^*p*_*k*_×*q*_*n*_^:

(2) $$\begin{array}{c} C=\underset{k\in Z_{n}}{⨁}C_{k}=\mathrm{diag}\left(\;C_{0},C_{1},\ldots ,C_{n-1}\;\right), \end{array}$$

where **C** ∈ *ℂ* ^*p*×*q*^, *p* = ∑_*j*∈*ℤ*_*n*__ *p*_*j*_, and *q* = ∑_*j*∈*ℤ*_*n*__ *q*_*j*_.

Let *n* = *rs*. The stride permutation matrix is defined as **L**_*s*_^*n*^ ∈ *ℂ* ^*n*×*n*^ such that it permutes the elements of the input signal **x** ∈ *ℂ* ^*n*^ as *jr* + *k* → *ks* + *j*, *j* ∈ *ℤ*_*s*_, and *k* ∈ *ℤ*_*r*_ [12, 14]. This matrix permutation governs the data flow required to parallelize a Kronecker product computation [12]. We clarify that the superscript *n* is an index, not power.

The vec operator, *𝒱* : *ℂ* ^*m*×*n*^ → *ℂ* ^*mn*^, transforms a matrix into a vector by stacking all the columns of this matrix one underneath the other. On the other hand, the vec inverse operator, *ℛ*_*m*,*n*_ : *ℂ* ^*mn*^ → *ℂ* ^*m*×*n*^, transforms a vector of dimension *mn* into a matrix of size *m* × *n*.

### 2.2. Discrete Fourier Transform

Let *l* ^2^(*ℤ*_*n*_) be the set of *ℂ*-valued signals on *ℤ*_*n*_; that is, **x** ∈ *l* ^2^(*ℤ*_*n*_) if and only if **x** ∈ *ℂ* ^*n*^ [9]. Additionally, for each *k*_1_ ∈ *ℤ*, **x**(*k*_1_) = **x**(*k*_2_), where *k*_2_ ∈ *ℤ*_*n*_ and *k*_1_ ≡ *k*_2_mod⁡*n*. The discrete Fourier transform (DFT) of **x** ∈ *l* ^2^(*ℤ*_*n*_) is represented as *ℱ*_**x**_ : *ℤ*_*n*_ → *ℂ* such that *ℱ*_**x**_(*k*) = ∑_*m*∈*ℤ*_*n*__ **x**(*m*)*ω*_*N*_^−*mk*^, where *ω*_*n*_ = exp⁡(2*πi*/*n*) and $i=\sqrt{-1}$.

As mentioned in [14], there are two different approaches of representing the DFT: as matrix-vector products or using summations. Consequently, fast algorithms using parallel computing are represented with either a matrix formalism as in [10, 12–14] or summations as in most signal processing books. Below, the matrix formalism is introduced and used to express the Cooley-Tukey FFT algorithm, corresponding to the decomposition of the transform size *n* into the product of two factors *r* and *s*; that is, *n* = *rs*.

The matrix representation of DFT of **x** is *ℱ*_**x**_ = **F**_*n*_ **x**, where **F**_*n*_ ∈ *ℂ* ^*n*×*n*^ such that **F**_*n*_(*j*, *k*) = *ω*_*N*_^−*jk*^. If *n* = *rs*, then the matrix formalism can be used to express **F**_*n*_ as factorizations of matrices using block matrices operations [10, 12, 13]:

(3) $$\begin{array}{c} F_{n}=L_{s}^{n}\left(\;I_{r}⊗F_{s}\;\right)L_{r}^{n}T_{r}^{n}\left(\;I_{s}⊗F_{r}\;\right)L_{s}^{n}. \end{array}$$

Here, **T**_*r*_^*n*^ is a diagonal matrix containing the twiddle factors. We clarify that the superscript *n* is an index, not power. This factorization of **F**_*n*_ is the matrix representation of the Cooley-Tukey FFT for *n* = *rs*. In addition, this representation of **F**_*n*_ allows the implementation using parallel computing [14].

## 3. DFT for Vector-Valued Signals

Based on [2, 6–9, 15, 16], the space of vector-valued discrete-time signals with *n* samples is defined as

(4) $$\begin{array}{c} l^{2}\left(\;Z_{n},C^{d}\;\right)=\left\{\;{\left(\;x_{0},x_{1},\ldots ,x_{n-1}\;\right)}^{T}:x_{j}\in C^{d},j\in Z_{n}\;\right\}. \end{array}$$

The space *l* ^2^(*ℤ*_*n*_, *ℂ* ^*d*^) is the set of *ℂ* ^*d*^-valued signals on *ℤ*_*n*_; that is, **x** ∈ *l* ^2^(*ℤ*_*n*_, *ℂ* ^*d*^) if and only if **x** ∈ *ℂ* ^*nd*^. Additionally, for each *k*_1_ ∈ *ℤ*, **x**_*k*_1__ = **x**_*k*_2__, where *k*_2_ ∈ *ℤ*_*n*_ and *k*_1_ ≡ *k*_2_mod⁡*n*. Furthermore, if *d* = 1, then *l* ^2^(*ℤ*_*n*_, *ℂ* ^*d*^) = *l* ^2^(*ℤ*_*n*_). Now, for **x** ∈ *l* ^2^(*ℤ*_*n*_, *ℂ* ^*d*^), there is a kind of DFT for vector-valued signals called* vector-valued DFT*. This transform is defined as *ℱ*_**x**_^*d*^ : *ℤ*_*n*_ → *ℂ* ^*d*^ such that

(5) $$\begin{array}{c} F_{x}^{d}\left(\;k\;\right)=\underset{m\in Z_{n}}{\sum }W_{n}^{-mk}x_{m}, \end{array}$$

where **W**_*n*_ ∈ *ℂ* ^*d*×*d*^ is the matrix kernel.  Algorithm 1 shows the implementation of (5). This implementation is a sequential algorithm.

From the reviewed literature, there are two kinds of kernels for this transform: the first one is* hypercomplex DFT kernel* [8]:

(6) $$\begin{array}{c} W_{n}=\exp\left(\;\frac{2\pi }{n}\cdot J\;\right)=\cos\left(\;\frac{2\pi }{n}\;\right)\cdot I_{d}+\sin\left(\;\frac{2\pi }{n}\;\right)\cdot J, \end{array}$$

where **J** ∈ *ℂ* ^*d*×*d*^ such that **J** ^2^ = −**I**_*d*_, and the second one is* DFT frame kernel* [7]:

(7) $$\begin{array}{c} W_{n}=\frac{1}{\sqrt{d}}\mathrm{diag}\left(\;\omega_{n}^{\alpha_{0}},\omega_{n}^{\alpha_{1}},\ldots ,\omega_{n}^{\alpha_{d-1}}\;\right), \end{array}$$

where *𝒜* = {*α*_0_, *α*_1_,…, *α*_*d*−1_} ⊂ *ℤ*_*n*_ with *α*_*j*_ < *α*_*k*_ for *j* < *k*. It is called DFT frame kernel because {**e**_*j*_}_*j*∈*ℤ*_*n*__ ⊂ *ℂ* ^*d*^, where $e_{j}=(1/\sqrt{d}){\left(\omega_{n}^{j\alpha_{0}},\omega_{n}^{j\alpha_{1}},\ldots ,\omega_{n}^{j\alpha_{d-1}}\right)}^{T}$ is a DFT frame. In this paper, subsets *𝒜* ⊂ *ℤ* ^+^ are used, such that card⁡(*𝒜*) = *d*, although it does not represent a DFT frame.

**Lemma 1 .**

Let **W**_*n*_ ∈ *ℂ* ^*d*×*d*^ be a hypercomplex DFT kernel or DFT frame kernel. Then

(1) **W**_*n*_^*j*+*r*^ = **W**_*n*_^*j*^ · **W**_*n*_^*r*^.
(2) **W**_*n*_^0^ = **W**_*n*_^*n*^ = **I**_*d*_.
(3) If *k* ∈ *ℤ* and *r* ∈ *ℤ*_*N*_, then **W**_*n*_^*nk*+*r*^ = **W**_*n*_^*r*^.
(4) If *n* = *rs*, then **W**_*n*_^*rk*^ = **W**_*s*_^*k*^.

**Proof**

For hypercomplex DFT kernel, the proof of each case is similar to proof of *n*th roots of unity. For DFT frame kernel, **W**_*n*_ is a diagonal matrix, and then the proof of each case is straightforward.

## 4. A Parallel Framework for *n*  =  *rs*

In this section, the main results of this paper are presented. Firstly, a block matrix representation of the vector-valued DFT is given. Secondly, a new mathematical framework from matrix representation of vector-valued DFT is derived, using a block matrix formalism (i.e., Theorem 2). This new result is inspired in the matrix representation of the Cooley-Tukey FFT algorithm for complex discrete-time signals, corresponding to the decomposition of the transform size *n* into the product of two factors *r* and *s*, which is developed in [10, 12, 13]. The result obtained in Theorem 2 is transformed in a new block matrix representation such that it contributes to analysis, design, and implementation of parallel algorithms (i.e., Corollary 3). This new result is inspired in (3). Finally, a computational complexity analysis of new algorithm is developed.

Similar to the DFT matrix representation explained in Section 2.2, there are two different approaches of representing the vector-valued DFT: as summations (see (5)) or using matrix-vector products. Both approaches allow a parallel implementation. In fact, the proof of Theorem 2 is developed using summation notation.

The vector-valued DFT can be presented as matrix-vector products. The block matrix representation of vector-valued DFT of **x** ∈ *l* ^2^(*ℤ*_*n*_, *ℂ* ^*d*^) is defined as *ℱ*_**x**_^*d*^ = **F**_*n*_^*d*^ **x**, where **F**_*n*_^*d*^ ∈ *ℂ* ^*dn*×*dn*^ such that (**F**_*n*_^*d*^)_*j*,*k*_ = **W**_*n*_^−*jk*^ ∈ *ℂ* ^*d*×*d*^, for *j*, *k* ∈ *ℤ*_*n*_. We clarify that the superscript *d* is an index, not power. In this section, a block matrix factorization of **F**_*n*_^*d*^ is developed, and it is inspired in (3). First, a generalization of stride permutation is defined. Let *n* = *rs*. The block stride permutation matrix [14, 17] is defined as **L**_*s*_^*n*,*d*^ ∈ *ℂ* ^*dn*×*dn*^ such that **L**_*s*_^*n*,*d*^ = **L**_*s*_^*n*^ ⊗ **I**_*d*_, and, for each **x** ∈ *ℂ* ^*dn*^ with *n* blocks **x**_*j*_ ∈ *ℂ* ^*d*^, the operation **L**_*s*_^*n*,*d*^ **x** permutes each block of the input block **x** as *jr* + *k* → *ks* + *j*, *j* ∈ *ℤ*_*s*_, and *k* ∈ *ℤ*_*r*_.

**Theorem 2 .**

Let *n* = *rs* and let **F**_*n*_^*d*^ ∈ *ℂ* ^*dn*×*dn*^ be the block matrix of DFT for vector-valued signals. Then

(8) $$\begin{array}{c} F_{n}^{d}=\left(\;F_{s}^{d}⊗I_{r}\;\right)T_{r}^{n,d}\left(\;I_{s}⊗F_{r}^{d}\;\right)L_{s}^{n,d}, \end{array}$$

where **T**_*r*_^*n*,*d*^ = ⨁_*j*∈*ℤ*_*s*__ **D**_*r*_^*j*^ such that **D**_*r*_ = ⨁_*k*∈*ℤ*_*r*__ **W**_*n*_^−*k*^.

**Proof**

Let **x** ∈ *ℂ* ^*dn*^, let *l*_1_, *k*_1_ ∈ *ℤ*_*r*_, and let *l*_2_, *k*_2_ ∈ *ℤ*_*s*_. The block vector **y** = (**I**_*s*_ ⊗ **F**_*r*_^*d*^)**L**_*s*_^*n*,*d*^ **x** is defined. Then

(9) $$\begin{array}{c} y_{k_{2}r+l_{1}}=\underset{k_{1}\in Z_{r}}{\sum }W_{r}^{-k_{1}l_{1}}x_{sk_{1}+k_{2}}. \end{array}$$

Now, let **z** = **T**_*r*_^*n*,*d*^ **y**. From Lemma 1, **W**_*r*_^−*k*_1_*l*_1_^ = **W**_*n*_^−*sk*_1_*l*_1_^; then

(10) zk2r+l1Wn−k2l1yk2r+l1=Wn−k2l1∑k1∈ZrWn−sk1l1xsk1+k2=∑k1∈ZrWn−sk1l1+k2l1xsk1+k2.

Let **w** = (**F**_*s*_^*d*^ ⊗ **I**_*r*_)**z**. Then

(11) wl1+l2r∑k2∈ZsWs−k2l2zk2r+l1=∑k2∈ZsWs−k2l2∑k1∈ZrWn−sk1l1+k2l1xsk1+k2=∑k2∈ZsWn−rk2l2∑k1∈ZrWn−sk1l1+k2l1xsk1+k2=∑k2∈Zs∑k1∈ZrWn−rk2l2+sk1l1+k2l1xsk1+k2.

But *rk*_2_ *l*_2_ + *sk*_1_ *l*_1_ + *k*_2_ *l*_1_ ≡ (*k*_2_ + *k*_1_ *s*)(*l*_1_ + *l*_2_ *r*)mod⁡*n*; then

(12) wl1+l2r∑k2∈Zs∑k1∈ZrWn−k2+k1sl1+l2rxsk1+k2= ∑k2∈Zs ∑k1∈ZrWn−k2+k1sl1+l2rxsk1+k2.

Let *m* = *sk*_1_ + *k*_2_, let *k* = *l*_1_ + *l*_2_ *r*, and let *m*, *k* ∈ *ℤ*_*n*_ because *l*_1_, *k*_1_ ∈ *ℤ*_*r*_, *l*_2_ *k*_2_ ∈ *ℤ*_*s*_, and *n* = *rs*. Then

(13) $$\begin{array}{c} \underset{m\in Z_{n}}{\sum }W_{n}^{-mk}x_{m}=F_{x}^{d}\left(\;k\;\right). \end{array}$$

Now, if *n* = *rs*, **A** ∈ *ℂ* ^*r*×*r*^, and **B** ∈ *ℂ* ^*ds*×*ds*^, the following equality [17] is obtained:

(14) $$\begin{array}{c} B⊗A=L_{s}^{n,d}\left(\;A⊗B\;\right)L_{r}^{n,d}. \end{array}$$

From Theorem 2 and (14), the following corollary presents a matrix factorization of **F**_*n*_^*d*^ such that it permits an implementation using parallel computing.

**Corollary 3 .**

Let *n* = *rs* and let **F**_*n*_^*d*^ ∈ *ℂ* ^*dn*×*dn*^ be the block matrix of DFT for vector-valued signals. Then

(15) $$\begin{array}{c} F_{n}^{d}=L_{s}^{n,d}\left(\;I_{r}⊗F_{s}^{d}\;\right)L_{r}^{n,d}T_{r}^{n,d}\left(\;I_{s}⊗F_{r}^{d}\;\right)L_{s}^{n,d}, \end{array}$$

where **T**_*r*_^*n*,*d*^ was defined in Theorem 2.

Algorithm 2 shows a parallel implementation of (15).

**Figure 1:**
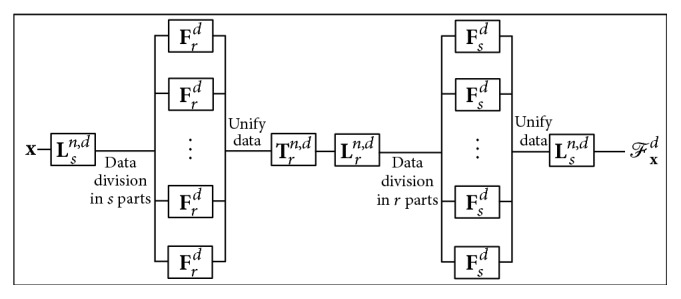
Parallel model of vector-valued DFT for **x** ∈ *l* ^2^(*ℤ*_*n*_, *ℂ* ^*d*^), *n* = *rs*, using a matrix representation.

*r* independent processes in Steps (3)–(5), and 2*s* independent processes in Steps (6)–(8) and (12)–(14) are observed, making this approach a parallel operation. A model of Algorithm 2 is shown in Figure 1.

### 4.1. Computational Complexity Analysis

In this section, the computational complexity analysis of (15) is developed. First, consider the matrix operation **L**_*s*_^*n*,*d*^ **v**. The computational complexity (CC) of **L**_*s*_^*n*,*d*^ **v** is *𝒪*(*nd*) [8] because it is the multiplication between a block matrix in *ℂ* ^*dn*×*dn*^ and a block vector in *ℂ* ^*dn*^. But the operation **L**_*s*_^*n*,*d*^ **v** can be implemented with a CC *𝒪*(*sd*) (see, e.g., [12, 14]).

Let **F**_*n*_^*d*^ ∈ *ℂ* ^*dn*×*dn*^ be the block matrix and vector-valued signal **x** ∈ *l* ^2^(*ℤ*_*n*_, *ℂ* ^*d*^), where *n* = *rs*. It is known that the CC of operation **y** = **F**_*n*_^*d*^ **x** is *𝒪*(*n* ^2^ *d* ^2^) = *𝒪*(*r* ^2^ *s* ^2^ *d* ^2^). Now consider operation **y** = **F**_*n*_^*d*^ **x** using (15). If we consider each matrix-vector multiplication, we obtain the following:

(1) The CC of **y**_1_ = **L**_*s*_^*n*,*d*^ **x** is *𝒪*(*sd*).
(2) The CC of **y**_2_ = (**I**_*s*_ ⊗ **F**_*r*_^*d*^)**y**_1_ is *𝒪*(*sr* ^2^ *d* ^2^), because it is a block diagonal matrix multiplication.
(3) The CC of **y**_3_ = **T**_*r*_^*n*,*d*^ **y**_2_ is *𝒪*(*nd*), because **T**_*r*_^*n*,*d*^ is a diagonal matrix multiplication.
(4) The CC of **y**_4_ = **L**_*r*_^*n*,*d*^ **y**_3_ is *𝒪*(*rd*).
(5) The CC of **y**_5_ = (**I**_*r*_ ⊗ **F**_*s*_^*d*^)**y**_4_ is *𝒪*(*rs* ^2^ *d* ^2^), because it is a block diagonal matrix multiplication.
(6) The CC of **y** = **L**_*s*_^*n*,*d*^ **y**_5_ is *𝒪*(*sd*).

Therefore, the CC of **F**_*n*_^*d*^ **x** using (15) is

(16) Osd+Osr2d2+Ond+Ord+Ors2d2+Osd=Osrr+sd2.

Thus, the CC of operation **F**_*n*_^*d*^ **x** is *𝒪*(*r* ^2^ *s* ^2^ *d* ^2^) and the CC of operation **F**_*n*_^*d*^ **x** using (15) is *𝒪*(*sr*(*r* + *s*)*d* ^2^). The above mentioned shows the efficiency of matrix formulation in (15).

## 5. Implementation and Experimental Investigation

### 5.1. General Information

The investigations have been carried out on a computer with multicore processor. The computer consists of 4 cores with Intel Core i7-3632QM CPU processor, system clock of 2.20 GHz, and 8 GB of RAM. The experiment develops the implementation and testing of Algorithms 1 and 2 with the hypercomplex DFT kernel and the DFT frame kernel is developed.  Algorithm 1 does not use any parallel implementation, unlike Algorithm 2. A CAZAC signal in *l* ^2^(*ℤ*_*n*_, *ℂ* ^*d*^) is used; it is generated using a Wiener CAZAC signal in *l* ^2^(*ℤ*_*n*_) [9] with *d* = 5 and *n* = *rs*, where *n* = 1024 = 32 · 32, *n* = 2048 = 64 · 32, *n* = 4096 = 64 · 64, *n* = 8192 = 128 · 64, and *n* = 16384 = 128 · 128.

The implementation of Algorithms 1 and 2 to compute the vector-valued DFT is performed using MATLAB. Algorithm 2 is computed using Parallel Computing Toolbox. MATLAB uses built-in multithreading and parallelism using MATLAB workers. Parallelism using MATLAB workers is used. We can run multiple MATLAB workers (MATLAB computational engines) on a multicore computer to execute applications in parallel with the Parallel Computing Toolbox. This approach allows more control over the parallelism compared to built-in multithreading. With programming constructs, such as parallel-for-loops (parfor) and batch, we write the parallel MATLAB programs of the parallel framework for the vector-valued DFT.

### 5.2. Results and Discussion

Let *T*_*∗*_ be the execution time of Algorithm 1 without any parallel implementation, and let *T*_*p*_ be the execution time of Algorithm 2, where *p* is the number of cores. The value of *T*_*p*_ needs to be less than that of *T*_*∗*_ for two reasons: Algorithm 2 has a parallel implementation and the matrix multiplication size is different. Algorithm 2 is computed with matrices in *ℂ* ^*dr*×*dr*^ and *ℂ* ^*ds*×*ds*^.  Algorithm 1 is computed with matrices in *ℂ* ^*dn*×*dn*^, where *n* = *rs*.

The computational performance analysis of Algorithm 2 is evaluated using the metrics speedup (or acceleration) and efficiency. The speedup is the ratio between the execution times of parallel implementations with one core and parallel implementations with two or more cores [18]. The speedup is represented by the formula *S* = *T*_1_/*T*_*p*_. The efficiency estimates how well utilized the processors are in solving the problem compared to how much effort is wasted in communication and synchronization [18]. The efficiency is determined by the ratio between the speedup and the number of processing elements, represented by the formula *E* = *T*_1_/(*pT*_*p*_).

Table 1 shows the execution time, in seconds (s), of both algorithms. A significant reduction in the parallel execution time of the vector-valued DFT is observed. Table 1 shows that Algorithm 1 with hypercomplex kernel for a Wiener CAZAC signal in *l* ^2^(*ℤ*_8192_, *ℂ* ^5^) produces a time of serial execution *T*_*∗*_ = 13408 s. Using Algorithm 2, however, we obtain *T*_1_ = 106.7 (0.80% of *T*_*∗*_), *T*_2_ = 80.44 s (0.60% of *T*_*∗*_), *T*_3_ = 57.35 s (0.43% of *T*_*∗*_), and *T*_4_ = 32.67 s (0.24% of *T*_*∗*_). This result shows the advantage of using multicore processors and a parallel computing environment to minimize the high execution time in the vector-valued DFT. This is because parallel computing is a form of computation in which many calculations are carried out simultaneously [19, 20], operating on the principle that large problems can often be divided into smaller ones, which are then solved concurrently, and minimize the execution time [20, 21]. The difference between *T*_*∗*_ and *T*_*p*_ is because *T*_*p*_ is computed with matrices in *ℂ* ^*dr*×*dr*^ and *ℂ* ^*ds*×*ds*^.  Algorithm 1 is computed with matrices in *ℂ* ^*dn*×*dn*^, where *n* = *rs*.

Table 2 represents the speedup of Algorithm 2. The acceleration of the vector-valued DFT increases when *p* increases regardless of the value of *n*. The results show that, using the proposed parallel implementation with *p* cores, where *p* = 2,3, 4, the speedup to compute the vector-valued DFT of a Wiener CAZAC signal is 1.09, 1.47, and 2.99, respectively. These results imply that, to get the highest speedup, one should prefer the approach with four cores.

Table 3 represents efficiency of Algorithm 2. The information in this table shows that a good efficiency (greater than 65%) is reached with *p* = 2. But the efficiency of the vector-valued DFT decreases (until 36%) when *p* increases regardless of the value of *n*. It is attributed to a decrease in the share of simultaneous computation of the partial vector-valued DFT in Algorithm 2 (steps (3)–(5) and (12)–(14)), which is responsible for the main effect. The results obtained in Table 3 imply that, to get a better efficiency, one should prefer the approach with two cores, because we obtain the highest efficiency.

## 6. Conclusion

This work presented a parallel framework of vector-valued DFT for vector-valued discrete-time signals. This mathematical framework was inspired in the matrix representation of the Cooley-Tukey FFT algorithm for complex discrete-time signals, corresponding to the decomposition of the transform size *n* into the product of two factors *r* and *s*, which is developed in [10, 12]. It was expressed in (15) and Algorithm 2. This parallel framework was performed in terms of a matrix representation using a set of block matrix operations: Kronecker product, direct sum, stride permutation, vec operator, and vec inverse operator. These operations contributed to analysis, design, and implementation in parallel. Two kernels are used in the vector-valued DFT: hypercomplex DFT kernel and DFT frame kernel.

The experimental investigation indicated there are profit using MATLAB with the Parallel Computing Toolbox in a computer with multicore processors. First, there was advantage to use multicore processors and a parallel computing environment to minimize the high execution time (with hypercomplex DFT kernel, we obtained *T*_*∗*_ = 13408 s, *T*_1_ = 106.7, *T*_2_ = 80.44 s, *T*_3_ = 57.35 s, and *T*_4_ = 32.67 s). Second, speedup increased when *p* increased regardless of the value of *n*, and a good efficiency too was obtained when *p* = 2 (above 65%).

As future work, we would like to extend the proposed parallel framework to vector-valued discrete-time signals in *l* ^2^(*ℤ*_*n*_, *ℂ* ^*d*^), where *n* = 2^*k*^, using the idea of Pease algorithm for complex discrete-time signals [22]. Additionally, we would like to take advantage of more design tradeoffs of different approaches besides what have been shown in this paper, for example, the approach developed in [23].

## Figures and Tables

**Algorithm 1:**
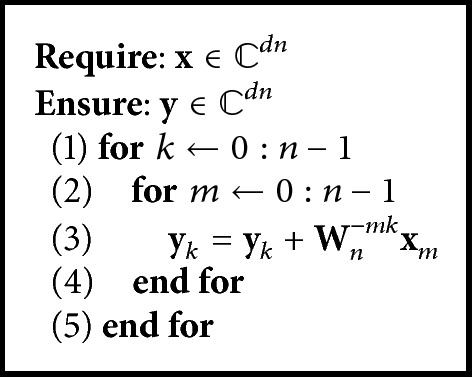
Vector-valued DFT (sequential algorithm).

**Algorithm 2:**
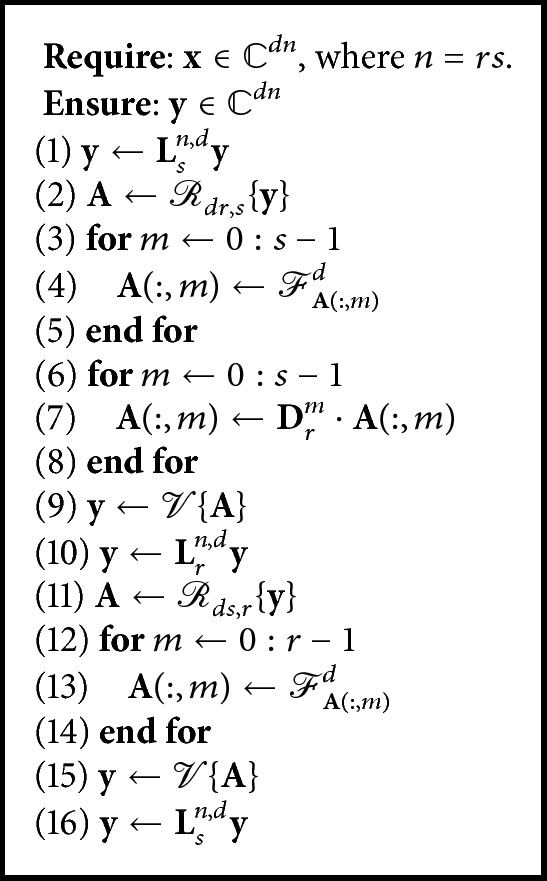
Vector-valued DFT (parallel algorithm).

**Table 1:** Computing time of Algorithms 1 and 2 (in seconds).

Kernel	*p*	*n*				
		1024	2048	4096	8192	16384
Hypercomplex DFT	*∗*	36.80	163.9	853.5	13408	+15000
	1	3.142	8.950	18.78	106.7	263.4
	2	2.363	4.276	17.25	80.44	180.9
	3	1.695	3.222	12.75	57.35	154.7
	4	0.983	2.966	6.481	32.67	82.65

DFT frame	*∗*	40.50	173.1	881.0	13913	+15000
	1	2.438	9.749	20.97	95.29	251.6
	2	1.911	5.798	16.38	58.72	179.5
	3	1.531	5.126	12.33	57.40	151.4
	4	1.199	2.329	5.366	31.46	73.42

Test signal in *l* ^2^(*ℤ*_*n*_, *ℂ* ^*d*^), where *n* = *rs* and *d* = 5.

*p* = *∗* is time execution of Algorithm 1.

*p* > 0 is the number of cores.

**Table 2:** Speedup of Algorithm 2.

Kernel	*p*	*n*				
		1024	2048	4096	8192	16384
Hypercomplex DFT	2	1.333	2.093	1.089	1.326	1.456
	3	1.853	2.778	1.473	1.860	1.703
	4	3.196	3.017	2.987	3.265	3.187

DFT frame	2	1.275	1.509	1.281	1.623	1.402
	3	1.592	1.707	1.701	1.660	1.661
	4	2.033	3.757	3.901	3.029	3.426

Test signal in *l* ^2^(*ℤ*_*n*_, *ℂ* ^*d*^), where *n* = *rs* and *d* = 5.

*p* is the number of cores.

**Table 3:** Efficiency of Algorithm 2.

Kernel	*p*	*n*				
		1024	2048	4096	8192	16384
Hypercomplex DFT	2	0.665	1.046	0.544	0.663	0.728
	3	0.463	0.694	0.368	0.465	0.426
	4	0.400	0.377	0.362	0.408	0.398

DFT frame	2	0.637	0.755	0.645	0.811	0.701
	3	0.530	0.427	0.425	0.415	0.415
	4	0.508	0.470	0.489	0.379	0.428

Test signal in *l* ^2^(*ℤ*_*n*_, *ℂ* ^*d*^), where *n* = *rs* and *d* = 5.

*p* is the number of cores.
